# A Tri-Oceanic Perspective: DNA Barcoding Reveals Geographic Structure and Cryptic Diversity in Canadian Polychaetes

**DOI:** 10.1371/journal.pone.0022232

**Published:** 2011-07-14

**Authors:** Christina M. Carr, Sarah M. Hardy, Tanya M. Brown, Tara A. Macdonald, Paul D. N. Hebert

**Affiliations:** 1 Biodiversity Institute of Ontario, University of Guelph, Guelph, Ontario, Canada; 2 University of Alaska, School of Fisheries and Ocean Sciences, Fairbanks, Alaska, United States of America; 3 Institute of Ocean Sciences, Fisheries and Oceans Canada, Sidney, British Columbia, Canada; J. Craig Venter Institute, United States of America

## Abstract

**Background:**

Although polychaetes are one of the dominant taxa in marine communities, their distributions and taxonomic diversity are poorly understood. Recent studies have shown that many species thought to have broad distributions are actually a complex of allied species. In Canada, 12% of polychaete species are thought to occur in Atlantic, Arctic, and Pacific Oceans, but the extent of gene flow among their populations has not been tested.

**Methodology/Principal Findings:**

Sequence variation in a segment of the mitochondrial cytochrome *c* oxidase I (COI) gene was employed to compare morphological versus molecular diversity estimates, to examine gene flow among populations of widespread species, and to explore connectivity patterns among Canada's three oceans. Analysis of 1876 specimens, representing 333 provisional species, revealed 40 times more sequence divergence between than within species (16.5% versus 0.38%). Genetic data suggest that one quarter of previously recognized species actually include two or more divergent lineages, indicating that richness in this region is currently underestimated. Few species with a tri-oceanic distribution showed genetic cohesion. Instead, large genetic breaks occur between Pacific and Atlantic-Arctic lineages, suggesting their long-term separation. High connectivity among Arctic and Atlantic regions and low connectivity with the Pacific further supports the conclusion that Canadian polychaetes are partitioned into two distinct faunas.

**Conclusions/Significance:**

Results of this study confirm that COI sequences are an effective tool for species identification in polychaetes, and suggest that DNA barcoding will aid the recognition of species overlooked by the current taxonomic system. The consistent geographic structuring within presumed widespread species suggests that historical range fragmentation during the Pleistocene ultimately increased Canadian polychaete diversity and that the coastal British Columbia fauna played a minor role in Arctic recolonization following deglaciation. This study highlights the value of DNA barcoding for providing rapid insights into species distributions and biogeographic patterns in understudied groups.

## Introduction

Molecular tools are increasingly recognized as necessary for delineating species boundaries, quantifying diversity, and clarifying distributions in understudied groups [1]–[3]. These tools can be particularly useful in marine systems where there is a poor understanding of species boundaries and broad-scale distributions. This lack of understanding is driven by the assumption that there are few barriers to gene flow and thus many ubiquitous species [4], [5], coupled with the reliance on morphological differences for species recognition [6]. The relatively recent detection of sibling species with restricted ranges, detectable only with molecular tools, supports the use of an integrative taxonomic approach to species delineation and range determination in ocean environments [6], [7].

Polychaetes are an abundant and speciose group in marine systems yet they are little studied compared to other taxa of similar ecological importance [8]. Despite their ubiquity (over 10,000 described species [9]–[11]), polychaetes have generally been excluded from broad-scale distributional studies because of their supposed lack of geographic structure [12]–[14]. In addition, traditional taxonomic approaches often result in the lumping of phenotypically similar species [15]. Consequently, there are substantial gaps in our knowledge of broad-scale diversity and distribution patterns. However, studies reporting biogeographic structure in polychaetes are becoming more frequent (e.g. along the Chilean coast [16], [17] and in the Mediterranean and Black Seas [18]) supporting similar investigation at even broader scales in other regions. Moreover, there is growing evidence that “widespread” polychaete species are more often a reflection of the limitations of conventional taxonomy than actual cosmopolitanism, which has led to an increased interest specifically in using genetic data to study taxon distributions [1], [19]–[23].

In Canadian marine waters, nearly 13% of the 1200 known polychaete species are thought to occur in all three oceans [24], but the extent of gene flow among their populations has not been tested. Another 35 species have been recorded in Atlantic and Pacific waters, but not in the Arctic (i.e. have amphiboreal distributions); however, the maintenance of gene flow between modern amphiboreal populations is questionable. In fact, it is possible that species with such disjunct distributions have been isolated ever since their migration through an ice-free Arctic over three million years ago (Ma) [25]. Certainly, extensive genetic structuring has been found in other marine taxa from this region, linked to vicariance caused by the repeated disruption of habitats and dispersal corridors throughout the Pleistocene [26]–[28]. These factors collectively suggest that a large-scale genetic survey of polychaete species across Canada will reveal overlooked species and shed light on the historic events that shaped modern distributions.

It is now recognized that traditional taxonomic approaches often overlook polychaete species [1] or are confused by colour polymorphisms [29], [30]. To address this limitation, several recent taxon-focused studies have examined variation in mitochondrial DNA (mtDNA) sequences and demonstrated that such analysis is valuable for the discrimination of closely related polychaete species [e.g. 15], [22], [29], [31]–[33]. Studies employing molecular data have consistently split presumed cosmopolitan species into complexes of often very divergent sibling species that have subsequently been found to show variation in other traits (e.g. *Eurythoe complanata* [15]; *Syllis gracilis* [19]). The DNA barcode initiative aims to catalogue global biodiversity by linking sequence data to voucher specimens [34], [35]. The barcode region for the animal kingdom is a 650 base pair fragment of the mitochondrial cytochrome *c* oxidase subunit I (COI) gene. Barcoding has frequently been used to recognize provisional species in groups with incomplete taxonomy [e.g. 36], [37], and morphological, ecological, and behavioural differences are regularly detected upon further examination of divergent taxa [e.g. 38], [39]. Barcoding has been particularly useful in marine systems [5] where variable reproductive forms and life stages, as well as damage to soft-bodied specimens during collection, hinder the identification of even well-known taxa [6]. Moreover, barcodes enable the rapid comparison of multiple taxa from widespread geographic regions and hold enormous potential for assessing genetic cohesion and for detecting broad-scale biogeographic patterns.

While several studies have suggested the efficacy of COI to discriminate polychaete species [e.g. 15], [29], [33], [40], [41], no broad genetic investigation, geographic or taxonomic, has been undertaken. In this study, we evaluate the utility of DNA barcoding for discriminating polychaete species, compare richness estimates based on morphological versus molecular taxonomy, examine the genetic structure of widespread species, and investigate broad-scale connectivity patterns among the oceans surrounding Canada.

## Results

### Amplification success

Three primer sets successfully amplified the barcode region of COI from 1876 of 2324 specimens (81% success). Initial polymerase chain reaction (PCR) with a new primer set polyLCO/polyHCO (Table S1) amplified approximately 70% of the individuals. Most species of the polychaete families Spionidae, Sabellidae, and Cirratulidae were amplified with a primer cocktail [42], while members of the family Nephtyidae were amplified with polyLCO/PolyshortCOIR (Table S1). Species of Serpulidae were amplified with polyLCO/polyHCO using PCR products as template for a second PCR. However, approximately 25 morphospecies, mostly serpulids, failed to yield amplicons suggesting residual difficulties in primer binding.

### COI genetic distances

**Figure 1:**
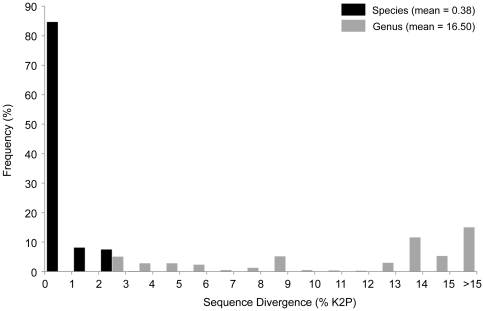
COI (K2P) distances for the barcode region within species and genera. Conspecific distances are based on provisional species assignments and congeneric values are between provisional species.

Mean COI Kimura two-parameter (K2P) sequence divergence between congeneric clusters was 40 times higher than within-cluster variation (16.50±0.05% and 0.38±0.01%, respectively) (Figure 1). Provisional species (molecular operational taxonomic units, or MOTUs) were identified using a two-step approach (see methods). A first pass screening using a 10× mean intracluster variation threshold of 3.8% suggested the presence of 315 provisional species. Haplotype network analysis of barcode clusters that were less than 3.8% divergent identified an additional 18 clusters as provisional species. In total, 333 provisional species representing 110 genera and 36 families resulted from analysis of 1876 specimens (Figure S1; Table S2). Presently, 194 MOTUs have been identified to a described species, 97 to a genus level, while the remaining 42 could only be assigned to a family level.

**Figure 2:**
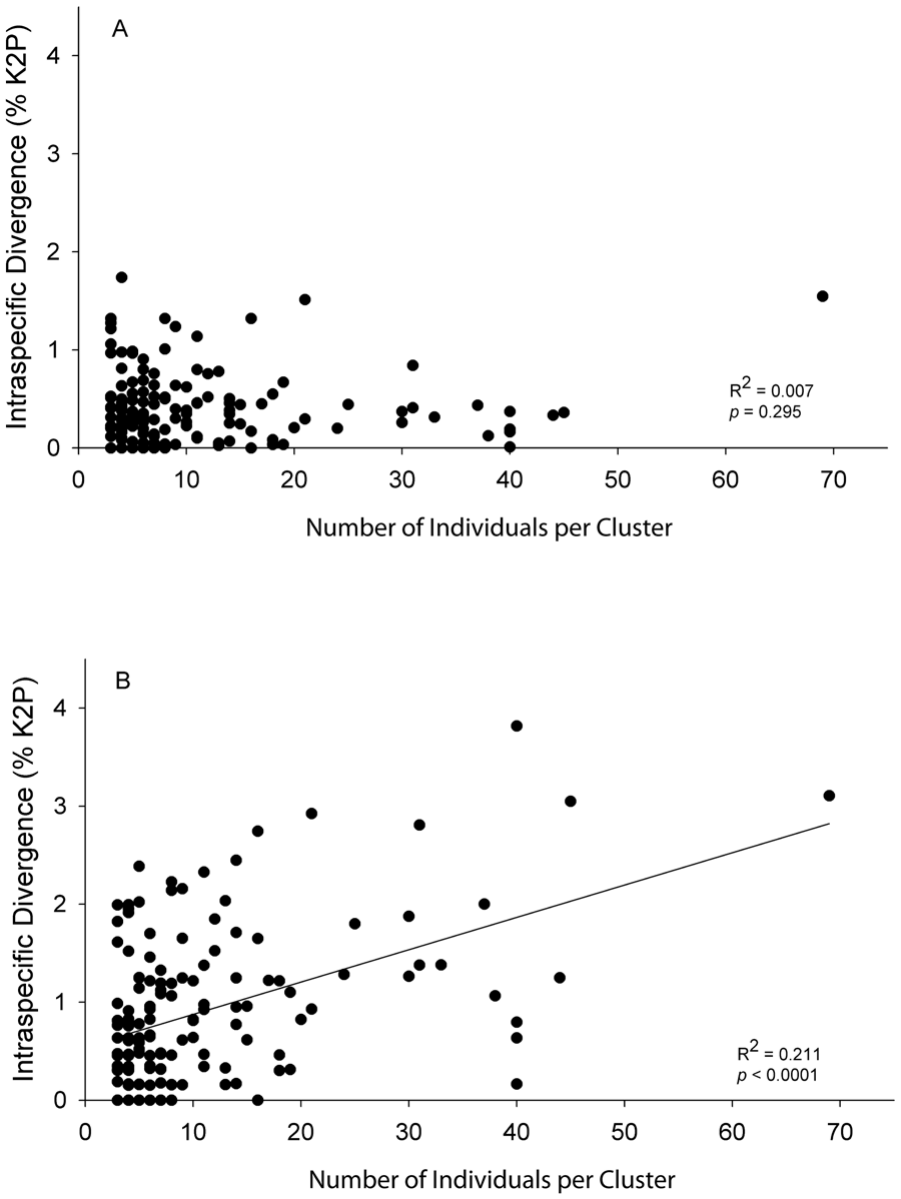
The effect of sample size on genetic distances. Linear regression of the number of individuals in each barcode cluster versus mean intraspecific distance (A) and maximum intraspecific distance (B).

**Figure 3:**
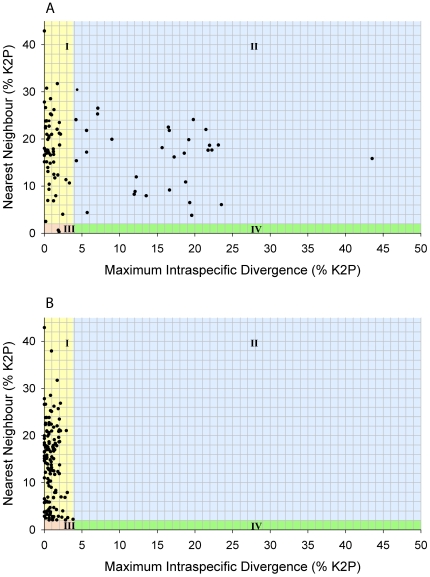
Comparison of COI (K2P) distances between morphologically identified polychaete species (A) and MOTUs (B). Plotting axes at 2% nearest neighbour divergence (y-axis) and 10× the average intracluster divergence of 3.8% (x-axis) creates four species categories where intraspecific distance and nearest neighbour distance respectively are: I) <3.8%, >2%: typical species; II) >3.8%, >2%: probable species complex; III) <3.8%, <2%: hybridization, young species, synonymy; IV) >3.8%, <2%: possible specimen misidentification.

The average number of individuals analyzed per MOTU was 5.7 (range of 1–69), but 119 of 333 were represented by a single specimen. The number of individuals analyzed per MOTU did not affect the average within-cluster variation (Figure 2A); however, maximum within-cluster variation did increase with the number of individuals analyzed per MOTU, though most divergences were under 3% (Figure 2B). Several morphologically identified species had intraspecific divergences of 15–25% (Figure 3A). These taxa always contained two or more distinct genetic clusters, which were partitioned into provisional species with maximum intracluster variation ranging from 0 to 3.8% (Figure 3B). On average, the maximum divergence within identified species was 5.9%. By contrast, the maximum K2P distance within MOTUs averaged 0.9%. Of the 194 pre-identified MOTUs, 106 had unique species assignments, while the remaining 88 provisional species derived from 34 identified species (Table 1) with an average intraspecific divergence of 12.5%. The commensal scale worms *Arctonoe fragilis* and *Arctonoe vittata* were the only two species to share a barcode cluster. Although intraspecific divergence in this cluster was relatively high (2.81%), the 16 specimens formed a connected parsimony network with no geographic or taxonomic division.

**Table 1:** Species with multiple MOTUs across Atlantic (AT), Arctic (AO), and Pacific (PC) Oceans of North America.

Species	No. MOTUs	% K2P	Lineages			Type locality
			AT	AO	PC	
*Alitta virens*	2	15.6	a		b	Norway, AT
*Barantolla americana*	2	22.9			a b	California, PC
*Brada villosa*	2	20.1		a	b	Norway, AT
*Capitella capitata*	2	21.8		a b		Greenland, AT
*Chone magna*	2	18.9			a b	California, PC
*Cirratulus cirratus*	3	18.2		a b	c	Europe, AT
*Eteone longa*	4	13.7		a b c d		Spitsbergen, AO
*Euclymene zonalis*	2	16.9	a b			Maine, AT
*Eumida minuta*	2	4.1		a b		Davis Strait, AO
*Eunoe nodosa*	2	2.6		a b		Norway, AT
*Eunoe oerstedi*	2	5.5	a	b		Sweden, AT
*Flabelligera affinis*	2	16.5		a b		Norway, AT
*Harmothoe imbricata*	6	11.6	a b	a b c d e	a e f	North Sea, AT
*Harmothoe rarispina*	3	10.2	a	a b	c	Norway, AT
*Laonice cirrata*	2	19.4			a b	Norway, AT
*Leitoscoloplos pugettensis*	5	15.9			a b c d e	Washington, PC
*Lepidonotus squamatus*	2	14.8	a		b	Europe, AT
*Lumbrineris fragilis*	2	3.0	a	b		Denmark, AT
*Myxicola infundibulum*	2	15.8	a		b	Medit. Sea, AT
*Naineris dendritica*	4	5.8			a b c d	British Columbia, PC
*Neoamphitrite robusta*	2	11.6			a b	Washington, PC
*Nephtys punctata*	3	8.5		a	b c	Alaska, PC
*Nereiphylla castanea*	2	22.7		a	b	Japan, PC
*Nereis pelagica*	3	3.7	a	b	b c	Europe, AT
*Nothria conchylega*	2	6.6		a	b	Norway, AT
*Ophelia limacina*	2	8.7		a b		Norway, AT
*Ophelina acuminata*	2	3.8	a	b		Denmark, AT
*Pectinaria granulata*	2	16.4	a	a	b	Europe, AT
*Phyllodoce groenlandica*	2	13.0		a b	b	Greenland, AT
*Praxillella praetermissa*	2	3.5		a b	a b	Norway, AT
*Scalibregma inflatum*	2	5.5		a	b	Norway, AT
*Syllis alternata*	5	26.7			a b c d e	Alaska, PC
*Syllis elongata*	2	4.2			a b	Washington, PC
*Terebellides stroemi*	4	20.3	a	b c d		Norway, AT

Each MOTU in an existing species is assigned a letter code (a-f). For cases in which species were split into more than two MOTUs, Kimura two-parameter (K2P) sequence divergence was averaged between all lineages.

### Morphological and molecular richness

**Figure 4:**
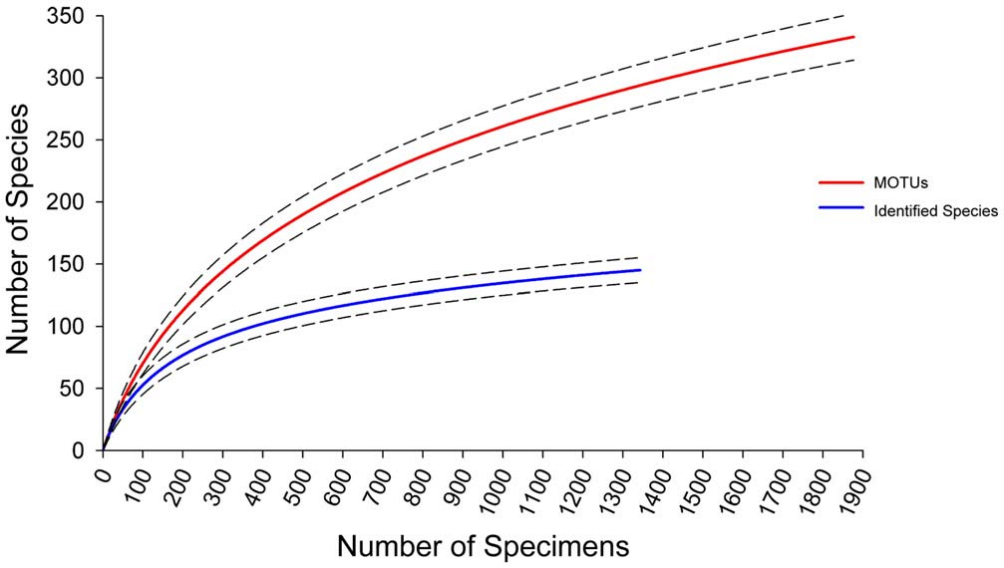
Morphological versus molecular diversity estimates. A comparison of rarefaction curves for morphologically identified species and MOTUs (95% confidence intervals are plotted).

Rarefaction curves were generated to compare species richness of morphological and molecular approaches at an equivalent sample size (Figure 4). The barcode curve showed a higher slope than the curve for identified species, with significant divergence (non-overlapping 95% confidence intervals) apparent after 200 specimens were sampled. When the richness of the two curves were compared at the minimum total sample size (n = 1343 morphologically identified specimens), the estimated richness of provisional species was more than twice as high as that for morphologically defined species (295 and 145 species, respectively).

### Species distributions

**Figure 5:**
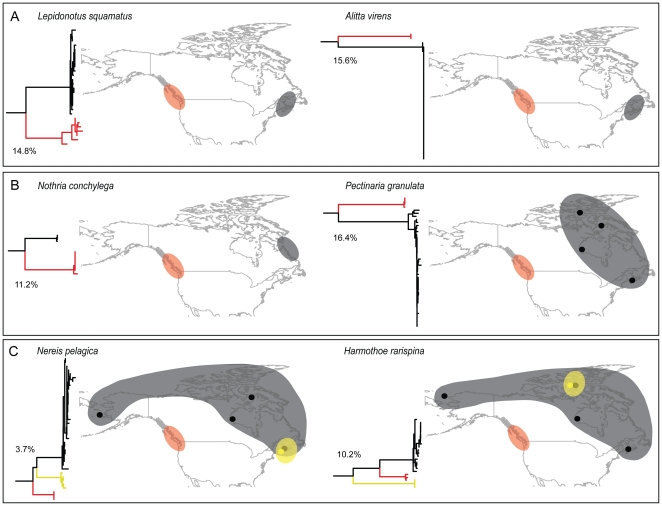
Examples of geographic splits in widespread species. Neighbour joining (NJ) trees with K2P divergences between amphiboreal species with divergent Pacific and Atlantic lineages (A), between species with divergent Pacific and Atlantic-Arctic lineages (B), and between species with multiple divergent lineages (K2P divergence is averaged among the three lineages) (C). Red, black, and yellow shaded regions correspond with branch colour on the NJ tree and indicate different lineages.

Pacific British Columbia had the highest incidence of provisional species that showed restricted distributions (i.e. were not shared among other sites; 98% of MOTUs), followed by the Arctic (71%), and Atlantic (62%), while the North Pacific (Bering Sea) had the lowest proportion of unique species (48%). Thirty-five percent of provisional species from the Atlantic had distributions extending into Arctic waters. By contrast, only 2% of provisional species from British Columbia had ranges that extended into the Arctic. No provisional species showed an amphiboreal distribution. Instead, Pacific and Atlantic populations of amphiboreal species always had more than 13% sequence divergence (Figure 5A; Table 1). Eighteen species with previously reported widespread distributions across Canada were collected from the Pacific and either or both the Atlantic and Arctic Oceans. Fouteen of these species were split into provisional species with large genetic divergences between Pacific and Atlantic-Arctic lineages (Figure 5B; Table 1) and five species had multiple divergent lineages (Figure 5C; Table 1). Eight provisional species had distributions spanning from the Bering Sea to the Eastern Arctic or Atlantic, but only one, *Harmothoe imbricata CMC01*, showed a continuous amphiboreal-arctic range extending from British Columbia to New Brunswick (Table 1).

### Faunal connectivity between oceans

**Figure 6:**
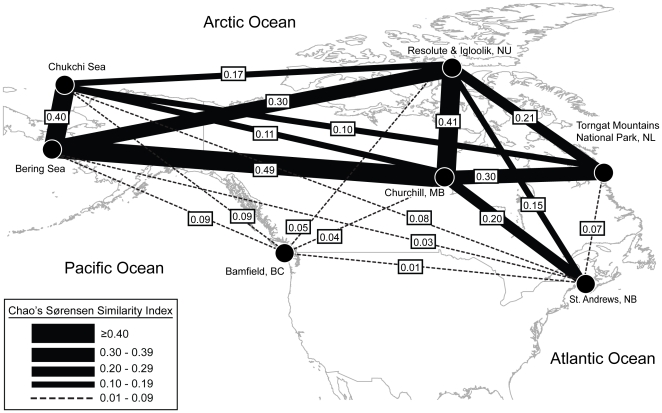
Faunal connectivity among sampling sites. The similarity in species composition among seven regions measured by Chao's Abundance-based Sørensen similarity index. Similarity values indicated in boxes (range 0–1, with 1 indicating complete overlap in species composition) are represented by line width between two regions.

The highest similarity in species composition occurred between Churchill and the Bering Sea (0.49) followed by Churchill and Nunavut (0.41), while the lowest congruence was between British Columbia and all other sites (mean = 0.05; Figure 6). Connectivity between sites in the Arctic Ocean and North Pacific (Bering Sea) was nearly seven times higher than connectivity between Arctic and boreal Pacific (British Columbia) collection sites (mean = 0.40 versus 0.06, respectively). Comparing the Arctic with boreal faunas of Atlantic (New Brunswick) and Pacific (British Columbia) basins, overlap was two times higher with the Atlantic (mean = 0.10 versus 0.05, respectively) (Figure 6). The lowest within-basin connectivity occurred in the Pacific, between British Columbia and Bering Sea sampling sites (0.09; Figure 6).

## Discussion

The present study demonstrates the effectiveness of DNA barcoding as a tool for species identification in polychaetes. The clustering pattern of COI barcodes flagged misidentifications, guided taxonomic decisions, and facilitated the detection of diversity overlooked by the current taxonomic system. Of the 142 species morphologically identified in this study, 34 contained multiple lineages representing nearly three times as many provisional species. This result suggests that broad application of DNA barcoding will accelerate the recognition and subsequent description of many currently undescribed polychaete species. Additionally, barcodes enabled the rapid assessment of species distributions, highlighted biogeographic trends, and provided insight into the events that have shaped the contemporary distributions and biodiversity of Canadian polychaetes.

### A DNA barcode reference library for Canadian polychaetes

This study involved the analysis of nearly 2000 specimens, representing 333 provisional polychaete species. Taking into account the 2∶1 ratio of provisional to identified species detected in this study (Figure 4), about 14% of the 1200 known Canadian species [24] have been surveyed. The Arctic was the most heavily sampled region with 140 provisional species (13% of known fauna), followed by the Pacific (120 provisional species, 8% of known fauna), and the Atlantic (86 provisional species, 9% of known fauna). Within the BOLD (Barcode of Life Data Systems) [35] reference library, sequences can be matched to 106 species supported by morphology and barcodes, 34 identified species representing 88 provisional barcode-supported species (e.g. *Pectinaria granulata CMC01*), and 97 genus-level provisional species (e.g. *Nephtys sp. CMC01*). Although several provisional species lack formal description, barcode results provide useful information about species' distribution and ecology as well as a framework for future taxonomic work [43]. Many of the species identified in this study are widespread throughout the Arctic and North Atlantic, suggesting the immediate utility of this newly populated sequence and voucher specimen library for the identification of specimens collected throughout these regions.

### Barcode clusters as provisional species

In most cases, the delineation of species boundaries from barcode data was straightforward. Although named species often included more than one provisional species, clusters were easily identifiable and typically highly divergent from other clusters. Barcode sequences differentiated all but one pair of morphologically identified polychaete species. The highest observed within-cluster divergence was 3.8%, indicating that barcodes naturally form tight clusters with low variation. Moreover, the 40-fold higher mean sequence divergence between than within clusters and the rarity of intermediate divergences (Figure 1) indicates that COI barcodes have high discriminatory power for polychaetes. For example, of the 333 recognized provisional species, only 18 showed <4% divergence from another cluster. However, this study also revealed sibling taxa that require more detailed investigation. For example, the maximum divergence within a lineage exceeded the minimum nearest-neighbour distance in two cases: *Harmothoe imbricata CMC04* (3.1% and 2.6%, respectively) and *Pholoe sp. CMC03* (2.8% and 2.1%). These patterns in variation might reflect the young age of recently isolated populations in North Atlantic and Arctic regions, which are known to harbour species with complex genetic patterning (e.g. *Macoma balthica*) [28]. Recent origins coupled with complex historical distributions make such young species particularly difficult to diagnose [7], [44].

Levels of between-species sequence divergence reported here are consistent with those found in other polychaete studies [e.g. 31]–[33], [45], [46]. Prior work has established that cryptic polychaete taxa discovered through genetic analysis regularly show correlated variation in other traits. For example, lineages of the *Polydora cornuta* complex [33] are reproductively isolated, members of the *Hediste japonica* complex show differences in life history [47], and sympatric sibling species of the *Polydora ciliata* complex show ecological differences [48]. Similar indications of ecological divergence were detected between provisional species delineated in this study. For example, habitat specialization was apparent in several taxa (e.g. Hudson Bay *Eteone* and *Ophelia*, British Columbia Syllidae). In other cases, high intraspecific divergences were detected between allopatric lineages (Figure 5; Table 1). Results suggest that populations of species assumed to have distributions spanning Canada's three oceans have gained reproductive isolation. This is likely a consequence of longstanding range fragmentation caused by cycles of glacial advance and retreat during the Pleistocene, which ultimately promoted diversification in northern marine taxa [e.g. 26]–[28]. While reproductive isolation is difficult to test among allopatric taxa [7], it can be inferred from molecular divergence between sympatric sibling taxa [49], or from persistent population structure in vicariant lineages following secondary contact [50]. Cases of both phenomena were apparent in this study. Divergent clusters within a species were evident in the absence of geographic barriers (e.g. British Columbia *Naineris dendritica*). Similarly, six lineages in the *Harmothoe imbricata* complex have come into secondary contact in the central Canadian Arctic, but remain distinct [51]. Whether these relatively young provisional species will continue on separate evolutionary trajectories or eventually merge as a result of introgressive hybridization requires further analysis of populations in zones of sympatry. Evidence for cryptic species in one geographic region suggests that a ‘phase two’ study linking previously undocumented diversity with ecological and historical correlates might reveal novel insight into mechanisms of speciation and historical phylogeography in polychaetes.

### Comparison of morphological and molecular species richness

Barcode-based species accumulation curves for Canadian polychaetes were considerably steeper than those based on morphologically delineated species (Figure 4). After analysis of 300 specimens, genetic species richness was 1.5 times higher than morphological species richness, and after analysis of 1000 specimens, richness estimates were doubled. Treating provisional species as functional units of biodiversity, these trends suggest that species richness in Canadian polychaetes is presently underestimated by a factor of at least two.

The effectiveness of DNA barcoding was originally established through work on groups where a strong taxonomic framework enabled tests of the validity of barcodes. The opportunity to critically examine whether COI-delineated species reflect morphological species in polychaetes occurred in the families Nereididae and Polynoidae, which were identified by taxonomic experts. In differentiating species, most disagreements between diagnostic methods reflected a conservative morphology. All identified Nereididae specimens were correctly assigned to species, no species shared a barcode, and divergences between species far exceeded those within. Barcodes further partitioned members of two species into geographically distinct lineages: the widespread *Nereis pelagica* (average 3.7% divergence), and *Alitta virens* (15.56%) (Figure 5; Table 1). Further study revealed morphological differences in the provisional Pacific species, *Alitta sp. CMC01*, which is currently under taxonomic investigation. Barcodes also flagged possible cryptic diversity in three species of Polynoidae: *Harmothoe imbricata*, *Harmothoe rarispina*, and *Lepidonotus squamatus*, all of which exhibited deep genetic divergences between Pacific and Atlantic lineages (Figure S1; Table 1). Additionally, in the Polynoidae, barcodes flagged two genetically indistinguishable species in the genus *Arctonoe*. Barcode sharing may arise in a variety of ways [52], but hybridization is possible since gametes of these species are highly compatible in reciprocal cross studies, suggesting their potential for gene exchange [53]. These species occur sympatrically and morphological intermediates were observed in this and other studies [53], [54]. Intermediate phenotypes are a general feature of hybrids [55], but may also indicate that *A. vittata* and *A. fragilis* constitute a single species with high phenotypic plasticity. Further examination with various nuclear genes should aid clarification of species boundaries in this genus.

### Species distributions and oceanic connectivity

A recent compilation of polychaete species recorded from Canada indicated that 148 species (12% of the fauna) occur in all three oceans surrounding Canada [24]. However, high divergence within several of these widespread species (Table 1) suggests that true cosmopolitanism may be much less common than previously reported. Most species with amphiboreal-arctic distributions examined in this study contained multiple provisional species with enough genetic divergence to designate them as provisional species (Figure 5; Table 1). Specifically, most species that transverse the Bering Strait with ranges extending from the Pacific to the Arctic or Atlantic only occur in the cold waters of the Bering Sea, and do not continue south into boreal British Columbia. Instead, large genetic discontinuities were consistently detected between British Columbia and Arctic or Atlantic populations, suggesting their long-term separation (Figure 5; Table 1). Connectivity patterns suggest that Bering Sea and Atlantic regions served as refugia during Pleistocene glaciations and that these faunas played a key role in Arctic recolonization following the retreat of ice. Conversely, the lack of similarity among coastal British Columbia and Arctic sites suggests that this fauna survived in a more southern Pacific refuge and did not contribute substantially to post-Pleistocene Arctic recolonization (Figure 6).

Divergences among lineages in this study may be attributed to historical range expansion through the Arctic Ocean via the Bering Strait and subsequent isolation when glaciers set in and water levels lowered. The northward flow of Pacific water through the Bering Strait provides the only natural connection between Pacific and Atlantic Oceans in the Northern Hemisphere [56]. Although many taxa likely originated from Pacific ancestors that migrated eastwards into the North Atlantic [27], [57], most were originally described from the Atlantic (Table 1) suggesting new names for Pacific lineages may be required. Consistent with other studies [e.g. 15], there was no evidence for contemporary gene flow among Atlantic and Pacific populations of amphiboreal species as no provisional species showed this type of distribution and all morphologically identified amphiboreal sister taxa were genetically distinct (Figure 5A; Table 1). Many temperate species that are thought to occur in boreal Atlantic and Pacific regions are likely distinct, reflecting their separation since a trans-Arctic migration 3.5 Ma [25], [58] or since the formation of ice sheets in North America 2.5–3 Ma [59]. The discovery of cases in which genetic data support the splitting of morphological species into multiple provisional species was anticipated, given the high incidence of cryptic species in polychaetes [1], [22], [60] and the complex glacial history of North America [25]–[27].

Consistent with other marine taxa [25], [58], the highest proportion of unique species was apparent in the Pacific (British Columbia), while Arctic and Atlantic species typically had broad distributions (Figure 6). Interestingly, the Atlantic had a lower proportion of unique species in this study than the Arctic, which may reflect its substantial impoverishment during the last glaciation and its recolonization by Pacific and European species [25], [58]. The lowest proportion of unique species was observed in the Bering Sea fauna. The Bering Sea region is a probable refuge for Pleistocene taxa [61], and the high connectivity with Arctic fauna suggests that this region was an important source for today's Arctic species. Indeed, many species in this study ranged from the Bering Sea through the Arctic and into the Atlantic. Furthermore, the highest overlap in species composition was observed between the Bering Sea and Hudson Bay. The Hudson Bay fauna is an impoverished subset of Arctic species [24], suggesting that many Hudson Bay polychaetes originated from the Bering Sea region. High connectivity between the Bering Sea and Arctic regions reflects recent or newly reestablished gene flow across the Bering Strait since its most recent opening *ca.*14,000 years ago [62] or periodically throughout the Pleistocene. Such recent gene flow between Pacific and Atlantic-Arctic waters has also been noted in echinoderms [63], algae [64], and molluscs [28].

This study corroborates the divergent nature of British Columbia and Bering Sea faunas first noted by Ushakov [65], who reported a biogeographic division between Pacific polychaetes on Asiatic and North American coasts. Considerable genetic structure in the Northeast Pacific has also been documented in the gastropod *Nucella lamellosa* [66] and the sea cucumber *Cucumaria pseudocurata* [67], suggesting Pleistocene survival in both northern and southern refugia [66], [67]. Ocean currents and temperature are thought to reinforce the break in North Pacific species distributions. For example, anticlockwise circulation of water in the Bering Sea and the porous Aleutian Island boundary may limit southward dispersal of Bering Sea species into the Gulf of Alaska [56]. Additionally, the faunal disconnect in the Pacific has been linked to the influx of Arctic waters into the North Pacific via the Bering Strait beginning 3–4 Ma [59], which promoted the division of boreal faunas on East and West Pacific coasts [57], [65]. Subsequent warming of coastal British Columbia may have forced cold water species north into the Bering Sea region [65]. Further sampling of populations between Arctic and boreal regions will aid in determining the approximate location of this well-supported biogeographic boundary in the Pacific.

### Conclusions

DNA barcoding provides an effective approach for the rapid evaluation of species richness in groups where many species await description. Furthermore, it enables the mapping of species distributions in the absence of formal taxonomic descriptions. The present investigation revealed the likely presence of many undescribed polychaete species in Canadian waters. This discovery was not unexpected since the number of polychaete species that await description is high. This study provides further support that DNA barcoding can accelerate the detection of overlooked species while creating a detailed taxonomic scaffold to aid future taxonomic research. Results of this study also support the assertion that far fewer polychaete species are as widely distributed as previously thought. Expanding the DNA reference library of northern hemisphere marine life holds enormous potential for detailed insights into broad-scale biogeographic patterns. This will ultimately lead to a better understanding of biodiversity, barriers to gene flow, and the process of speciation in ocean environments.

## Materials and Methods

### Specimens

A total of 2324 polychaetes were collected (detailed specimen information is available in the “Polychaetes of North America (PONA)” project on BOLD; www.barcodinglife.org [35]) between 1999 and 2009 from eight locations in Canada and Alaska. These sites included Bamfield (British Columbia; n = 553), Bering Sea (Alaska; n = 137), Chukchi Sea (Alaska; n = 26), Churchill (Manitoba; n = 864), Resolute (Nunavut; n = 167), Igloolik (Nunavut; n = 91), Torngat Mountains National Park (Newfoundland and Labrador; n = 77), and St. Andrews (New Brunswick; n = 409). Samples from most sites were collected in the intertidal zone or from nearshore coastal habitats using subtidal dredges, diving, and plankton tows. Bering and Chukchi Sea samples were obtained from ship-based sampling in offshore waters (40–60 m water depth) using a van Veen grab and a plumb-staff beam trawl with 4 mm mesh. Samples were sieved on 1 and 0.5 mm mesh. Whenever possible, multiple specimens of each morphospecies were collected. Specimens were fixed in either 70 or 90% ethanol, which was replaced at least three times to prevent dilution, and preserved in 95% ethanol.

Specimens were initially assigned to a family or genus using morphological criteria. Following DNA sequence analysis, specimens from each barcode cluster were morphologically identified using taxonomic keys for each region and, where possible, species identifications were verified by taxonomic specialists. Taxonomic assignments follow the World Register of Marine Species (WoRMS) [68]. In cases where a single species split into two or more distinct clusters, the Linnaean species name was retained and appended with interim identifiers (e.g. *Nereis pelagica CMC01*). Where the most precise identification was to a genus, similar interim names were applied (e.g. *Polycirrus sp. CMC01*). Morphological work is ongoing and taxonomic assignments will be updated as specimens are studied in more detail. Specimens will be deposited in the Canadian Museum of Nature and the Biodiversity Institute of Ontario. Collection data, photographs, specimen information, sequences, and trace files are available from the PONA project console on BOLD. Sequences have also been deposited in GenBank (accession nos. GU672096–GU672410, HM375142–HM375143, HM375485–HM375497, HM473288–HM473810, HM904906–HM904907, HM906747–HM906752, HQ023430–HQ023818, HQ023865–HQ024489).

### DNA isolation, extraction, and amplification

A small piece of muscle tissue was lysed in 45 µl cetyltrimethylammonium bromide (CTAB) lysis buffer solution [69] plus 5 µl proteinase K. These samples were incubated at 56°C for 12–18 hours and DNA was then extracted using the manual protocol of Ivanova et al. [69] with a 3 µm glass fibre plate and re-suspended in 50 µl of ddH_2_0. The target COI region was amplified using three primer sets (Table S1). PCR reactions were carried out in a 12.5 µl reaction volume containing 6.25 µl 10% trehalose, 2 µl ddH_2_0, 1.25 µl 10× PCR buffer, 0.625 µl MgCl_2_ (50 mM), 0.125 µl of each primer (10 µM), 0.0625 µl dNTPs (10 mM), 0.06 µl Platinum Taq polymerase, and 2 µl of DNA template (10–50 ng). The thermocycling profile for all primers (except the cocktail) consisted of one cycle of 1 min at 94°C, five cycles of 40 s at 94°C, 40 s at 45°C, and 1 min at 72°C, followed by 35 cycles of 40 s at 94°C, 40 s at 51°C, and 1 min at 72°C, with final extension for 5 min at 72°C. For the primer cocktail, thermocycling conditions were slightly modified [see 42]. PCR products were run on the E-Gel® 96-well system (Invitrogen) and PCR product was bidirectionally sequenced using BigDye v3.1 on an ABI 3730xl DNA Analyzer (Applied Biosystems).

Sequences were manually edited using Sequencher v4.5 (Gene Codes Corporation, Ann Arbor, MI) and aligned by ClustalW in MEGA 4.0 [70]. Anomalous sequences that were not obvious pseudogenes (i.e. no frameshifts, full-length) or contaminants (following BLAST-type searches) were re-analyzed with alternate primers to test for multiple copies of COI. Twelve species had multiple indels in their COI sequence (Serpulidae: n = 9, *Travisia*: n = 2, and *Spio setosa*), but in all cases the reading frame was preserved suggesting the amplified COI fragments are functional copies.

Barcode clusters were initially assigned provisional species status using a 10 times average intracluster divergence threshold [3], [44]. To further assess provisional species boundaries among clusters that were less divergent than this threshold, statistical parsimony networks were analyzed using the Templeton, Crandall, Sing parsimony algorithm (TCS) [71], [72]. This analysis constructs haplotype networks by inferring the most parsimonious branch connections at a 95% confidence level [72] and has been shown to accurately partition barcodes into species clusters [73]. Neighbour-joining (NJ) analysis using the K2P model [74] was conducted in MEGA 4.0 [70] to provide a graphical representation of the species tree, using one representative per MOTU. The K2P distance metric was chosen for consistency and comparability with other barcode studies and for its suitability when sequence divergences are low, such as for the construction of intra- and interspecies phylogenies [34], [75]. Moreover, the NJ K2P method is a computationally efficient approach that can be performed on the BOLD platform, making it well suited for large-scale and exploratory identification of provisional species using DNA barcodes. For all subsequent data analyses, MOTUs were used as proxies for species and genetic distances were calculated using the BOLD “Distance Summary” and “Nearest Neighbour Summary” tools [35].

### Regional species richness and similarity indices

To examine differences in molecular and morphological richness, MOTUs were compared to identified species in the cases where morphological designations were available. Individual-based rarefaction curves (S_obs_ Mao Tau) were generated separately for identified species (n = 1343) and for MOTUs (n = 1876) using the software EstimateS v.8.2.0 [76] with 50 randomizations and sampling without replacement. Diversity Data matrices were constructed as Species, Sample, Abundance triplets. To provide an overview of the similarity in species composition between collection sites, Chao's Abundance-based Sørensen similarity index [77], [78] was calculated. This shared species estimator modifies the classic Sørensen similiary index for presence-absence data by accounting for unequal or incomplete sampling of a region and is particularly useful when there are many rare species in a dataset [78]. Shared Species matrices were constructed in EstimateS [76] as Species, Sample, Abundance triplets with seven collection sites (samples) defined. Maps were obtained from SimpleMappr [79].

### Statistical analysis

To determine whether the number of individuals analyzed affected the level of variation within genetic clusters, a linear regression was performed. To test the assumptions of homoscedastic residual variance, a Spearman rank correlation was run on the absolute value of residual variation in K2P distance and the number of individuals sampled. A Shapiro-Wilk test was used to test if residuals were normally distributed, but no significant deviations were detected.

## Supporting Information

Figure S1 **Neighbour-joining tree for 333 provisional polychaete species.** One specimen per MOTU is shown with abundances indicated in brackets. Collection locations are indicated by pie graphs. Vertical bars indicate identified species whose members fall into two or more MOTUs.
(PDF)

Table S1 **List of primers used in this study.**
(PDF)

Table S2 **Polychaete family richness and abundance by sampling site.**
(PDF)

## References

[1] Westheide W, Schmidt H (2003). Cosmopolitan versus cryptic meiofaunal polychaete species: An approach to a molecular taxonomy.. Helgol Mar Res.

[2] Wörheide G, Solé-Cava AM, Hooper JNA (2005). Biodiversity, molecular ecology and phylogeography of marine sponges: Patterns, implications and outlooks.. Integr Comp Biol.

[3] Witt JDS, Threloff DL, Hebert PDN (2006). DNA barcoding reveals extraordinary cryptic diversity in an amphipod genus: Implications for desert spring conservation.. Mol Ecol.

[4] Palumbi SR (1994). Genetic divergence, reproductive isolation, and marine speciation.. Annu Rev Ecol Syst.

[5] Radulovici AE, Archambault P, Dufresne F (2010). DNA barcodes for marine biodiversity: Moving fast forward?. Diversity.

[6] Knowlton N (1993). Sibling species in the sea.. Annu Rev Ecol Syst.

[7] Knowlton N (2000). Molecular genetic analyses of species boundaries in the sea.. Hydrobiol.

[8] Quijón PA, Snelgrove PVR (2005). Polychaete assemblages of a sub-arctic Newfoundland fjord: Habitat, distribution, and identification.. Polar Biol.

[9] Hutchings P, Fauchald C, Beesley PL, Ross GJB, Glasby CJ (2000). Class Polychaeta. Definition and general description.. Polychaetes and allies: The southern synthesis.

[10] Jirkov IA (2001). Polychaeta of the Arctic Ocean.

[11] Rouse GW, Pleijel F (2001). Polychaetes.

[12] Fauchald K, Hutchings PA (1984). Polychaete distribution patterns, or: Can animals with Palaeozoic cousins show large-scale geographical patterns?. Proc 1^st^ Int Polychaete Conf.

[13] Pocklington P, Tremblay MJ (1987). Faunal zones in the northwest Atlantic based on polychaete distribution.. Can J Zool.

[14] Glasby CJ, Alvarez B (1999). Distribution patterns and biogeographic analysis of Austral Polychaeta (Annelida).. J Biogeogr.

[15] Barroso R, Klautau M, Solé-Cava AM, Paiva PC (2010). *Eurythoe complanata* (Polychaeta: Amphinomidae), the ‘cosmopolitan’ fireworm, consists of at least three cryptic species.. Mar Biol.

[16] Hernández CE, Moreno RA, Rozbaczylo N (2005). Biogeographical patterns and Rapoport's rule in southeastern Pacific benthic polychaetes of the Chilean coast.. Ecography.

[17] Moreno RA, Hernández CE, Rivadeneira MM, Vidal MA, Rozbaczylo N (2006). Patterns of endemism in south-eastern Pacific benthic polychaetes of the Chilean coast.. J Biogeogr.

[18] Arvanitidis C, Bellan G, Drakopoulos P, Valavanis V, Dounas C (2002). Seascape biodiversity patterns along the Mediterranean and the Black Sea: Lessons from the biogeography of benthic polychaetes.. Mar Ecol Prog Ser.

[19] Maltagliati F, Peru AP, Casu M, Rossi F, Lardicci C (2000). Is *Syllis gracilis* (Polychaeta: Syllidae) a species complex? An allozyme perspective.. Mar Biol.

[20] Bhaud MR, Petti MAV (2001). *Spiochaetopterus nonatoi*, a new species of Chaetopteridae (Polychaeta) from Brazil: Biogeographical consequences.. J Mar Biol Ass UK.

[21] Bhaud M, Koh BS, Martin D (2006). New systematic results based on chaetal hard structures in *Mesochaetopterus* (Polychaeta).. Sci Mar.

[22] Bleidorn C, Kruse I, Albrecht S, Bartolomaeus T (2006). Mitochondrial sequence data expose the putative cosmopolitan polychaete *Scoloplos armiger* (Annelida, Orbiniidae) as a species complex.. BMC Evol Biol.

[23] Martin D, Koh BS, Bhaud M, Dutrieux E, Gil J (2006). The genus *Owenia* (Annelida: Polychaeta) in the Persian Gulf, with description of *Owenia persica* sp. nov.. Org Div Evol.

[24] Carr CM (2011). Polychaete diversity and distribution patterns in Canadian marine waters.. Mar Biodiv.

[25] Vermeij GJ (1991). Anatomy of an invasion: The trans-Arctic interchange.. Paleobiol.

[26] Hobæk A, Weider LJ (1999). A circumpolar study of Arctic biodiversity: Phylogeographic patterns in the *Daphnia pulex* complex.. Ambio.

[27] Dodson JJ, Tremblay S, Colombani F, Carscadden JE, Lecomte F (2007). Trans-Arctic dispersals and the evolution of a circumpolar marine fish species complex, the capelin (*Mallotus villosus*).. Mol Ecol.

[28] Nikula R, Strelkov P, Väinölä R (2007). Diversity and trans-Arctic invasion history of mitochondrial lineages in the North Atlantic *Macoma balthica* complex (Bivalvia: Tellinidae).. Evolution.

[29] Nygren A, Pleijel F (2010). From one to ten in a single stroke – resolving the European *Eumida sanguinea* (Phyllodocidae, Annelida) species complex.. Mol Phyl Evol.

[30] Nygren A, Norlinder E, Panova M, Pleijel F (2011). Colour polymorphisms in the polychaete *Harmothoe imbricata* (Linnaeus, 1967).. Mar Biol Res.

[31] Glover AG, Goetze E, Dahlgren TG, Smith CR (2005). Morphology, reproductive biology and genetic structure of the whale-fall and hydrothermal vent specialist, *Bathykurila guaymasensis* Pettibone, 1989 (Annelida: Polynoidae).. Mar Ecol.

[32] Bastrop R, Blank M (2006). Multiple invasions - a polychaete genus enters the Baltic Sea.. Biol Inv.

[33] Rice SA, Stephen K, Rice KA (2008). The *Polydora cornuta* complex (Annelida: Polychaeta) contains populations that are reproductively isolated and genetically distinct.. Invert Biol.

[34] Hebert PDN, Cywinska A, Ball SL, deWaard JR (2003). Biological identifications through DNA barcodes.. Proc R Soc Lond B Biol Sci.

[35] Ratnasingham S, Hebert PDN (2007). BOLD: The Barcode of Life Data System http://www.barcodinglife.org.. Mol Ecol Notes.

[36] Floyd R, Abebe E, Papert A, Blaxter M (2002). Molecular barcodes for soil nematode identification.. Mol Ecol.

[37] Smith MA, Fisher BL, Hebert PDN (2005). DNA barcoding for effective biodiversity assessment of a hyperdiverse arthropod group: The ants of Madagascar.. Phil Trans R Soc B.

[38] Hebert PDN, Penton EH, Burns JM, Janzen DH, Hallwachs W (2004). Ten species in one: DNA barcoding reveals cryptic species in the neotropical skipper butterfly *Astraptes fulgerator*.. Proc Natl Acad Sci USA.

[39] Smith MA, Woodley NE, Janzen DH, Hallwachs W, Hebert PDN (2006). DNA barcodes reveal cryptic host-specificity within the presumed polyphagous members of a genus of parasitoid flies (Diptera: Tachinidae).. Proc Natl Acad Sci USA.

[40] Olson MA, Zajac RM, Russello MA (2009). Estuarine-scale genetic variation in the polychaete *Hobsonia florida* (Ampharetidae; Annelida) in Long Island Sound and relationships to Pleistocene glaciations.. Biol Bull.

[41] Pleijel F, Rouse G, Nygren A (2009). Five colour morphs and three new species of *Gyptis* (Hesionidae, Annelida) under a jetty in Edithburgh, South Australia.. Zool Script.

[42] Ivanova NV, Zemlak TS, Hanner RH, Hebert PDN (2007). Universal primer cocktails for fish DNA barcoding.. Mol Ecol Notes.

[43] Zhou X, Adamowicz SJ, Jacobus LM, DeWalt RE, Hebert PDN (2009). Towards a comprehensive barcode library for Arctic life – Ephemeroptera, Plecoptera, and Trichoptera of Churchill, Manitoba, Canada.. Front Zool.

[44] Hebert PDN, Stoeckle MY, Zemlak TS, Francis CM (2004). Identifications of birds through DNA barcodes.. PLoS Biol.

[45] Chevaldonne P, Jollivet D, Desbruyeres D, Lutz RA, Vrijenhoek RC (2002). Sister-species of eastern Pacific hydrothermal vent worms (Ampharetidae, Alvinellidae, Vestimentifera) provide new mitochondrial COI clock calibration.. Cah Biol Mar.

[46] Jolly MT, Jollivet D, Gentil F, Thiebaut E, Viard F (2005). Sharp genetic break between Atlantic and English Channel populations of the polychaete *Pectinaria koreni*, along the north coast of France.. Heredity.

[47] Sato M, Masuda Y (1997). Genetic differentiation in two sibling species of the brackish-water polychaete *Hediste japonica* complex (Nereididae).. Mar Biol.

[48] Manchenko GP, Radashevsky VI (1993). Genetic differences between two sibling species of the *Polydora ciliata* complex (Polychaeta: Spionidae).. Biochem Syst Ecol.

[49] Wayne RK (1992). On the use of morphologic and molecular genetic characters to investigate species status.. Cons Biol.

[50] Rohfritsch A, Borsa P (2005). Genetic structure of Indian scad mackerel *Decapterus russelli*: Pleistocene vicariance and secondary contact in the Central Indo-West Pacific Seas.. Heredity.

[51] Hardy SM, Carr CM, Hardman M, Steinke D, Corstorphine E (2011). Biodiversity and phylogeography of Arctic marine fauna: Insights from molecular tools.. Mar Biodiv.

[52] Kerr KCR, Stoeckle MY, Dove CJ, Weigt LA, Francis CM (2007). Comprehensive DNA barcode coverage of North American birds.. Mol Ecol Notes.

[53] Pernet B (1999). Gamete interactions and genetic differentiation among three sympatric polychaetes.. Evolution.

[54] Davenport D (1950). Studies in the physiology of commensalism. 1. The polynoid genus *Arctonoë*.. Biol Bull.

[55] Mallet J (2007). Hybrid speciation.. Nature.

[56] Stabeno PJ, Schumacher JD, Ohtani K, Loughlin TR, Ohtani K (1999). The physical oceanography of the Bering Sea.. Dynamics of the Bering Sea: A summary of physical, chemical, and biological characteristics, and a synopsis of research on the Bering Sea.

[57] Briggs JC (2007). Marine longitudinal biodiversity: Causes and conservation.. Diversity Distrib.

[58] Briggs JC (1970). A faunal history of the North Atlantic Ocean.. Syst Zool.

[59] Webb T, Bartlein PJ (1992). Global change during the last 3 million years: Climatic controls and biotic responses.. Annu Rev Ecol Syst.

[60] Bastrop R, Jurss K, Sturmbauer C (1998). Cryptic species in a marine polychaete and their independent introduction from North America to Europe.. Mol Biol Evol.

[61] Provan J, Bennett KD (2008). Phylogeographic insights into cryptic glacial refugia.. Trends Ecol Evol.

[62] Dunton K (1992). Arctic biogeography: The paradox of the marine benthic fauna and flora.. Trends Ecol Evol.

[63] Palumbi SR, Kessing BD (1991). Population biology of the trans-Arctic exchange: MtDNA sequence similarity between Pacific and Atlantic sea urchins.. Evolution.

[64] van Oppen MJH, Draisma SGA, Olsen JL, Stam WT (1995). Multiple trans-Arctic passages in the red alga *Phycodrys rubens*: Evidence from nuclear rDNAITS sequences.. Mar Biol.

[65] Ushakov PV (1965). Polychaeta of the far Eastern Seas of the USSR. Keys to the fauna of the USSR.

[66] Marko PB (2004). ‘What's larvae got to do with it?’ Disparate patterns of post-glacial population structure in two benthic marine gastropods with identical dispersal potential.. Mol Ecol.

[67] Arndt A, Smith MJ (1998). Genetic diversity and population structure in two species of sea cucumber: Differing patterns according to mode of development.. Mol Ecol.

[68] Fauchald K (2009). World Polychaeta database.. http://www.marinespecies.org.

[69] Ivanova NV, Fazekas AJ, Hebert PDN (2008). Semi-automated, membrane-based protocol for DNA isolation from plants.. Plant Mol Biol Rep.

[70] Tamura K, Dudley J, Nei M, Kumar S (2007). MEGA4: Molecular Evolutionary Genetics Analysis (MEGA) software version 4.0.. Mol Biol Evol.

[71] Templeton AR, Crandall KA, Sing CF (1992). A cladistic analysis of phenotypic associations with haplotypes inferred from restriction endonuclease mapping. III. Cladogram estimation.. Genetics.

[72] Clement M, Posada D, Crandall KA (2000). TCS: A computer program to estimate gene genealogies.. Mol Ecol.

[73] Hart MW, Sunday J (2007). Things fall apart: Biological species form unconnected parsimony networks.. Biol Lett.

[74] Kimura M (1980). A simple method for estimating evolutionary rate of base substitutions through comparative studies of nucleotide sequences.. J Mol Evol.

[75] Nei M, Kumar S (2000). Molecular evolution and phylogenetics.

[76] Colwell RK (2009). EstimateS: Statistical estimation of species richness and shared species from samples. Version 8.2.. http://purl.oclc.org/estimates.

[77] Sørensen TA (1948). A method of establishing groups of equal amplitude in plant sociology based on similarity of species content, and its application to analyses of the vegetation on Danish commons.. K Dan Vidensk Selsk Biol Skr.

[78] Chao A, Chazdon RL, Colwell RK, Shen TJ (2005). A new statistical approach for assessing similarity of species composition with incidence and abundance data.. Ecol Lett.

[79] Shorthouse DP (2010). SimpleMappr, a web-enabled tool to produce publication-quality point maps.. http://www.simplemappr.net.

