# Supplementary material for: A Tri-Oceanic Perspective: DNA Barcoding Reveals Geographic Structure and Cryptic Diversity in Canadian Polychaetes
Source: PLoS One. 2011 Jul 14;6(7):e22232. doi: 10.1371/journal.pone.0022232 (PMC3136506; doi:10.1371/journal.pone.0022232)
Supplement: Figure S1 — Neighbour-joining tree for 333 provisional polychaete species. One specimen per MOTU is shown with abundances indicated in brackets. Collection locations are indicated by pie graphs. Vertical bars indicate identified species whose members fall into two or more MOTUs. (PDF) [file pone.0022232.s001.pdf]

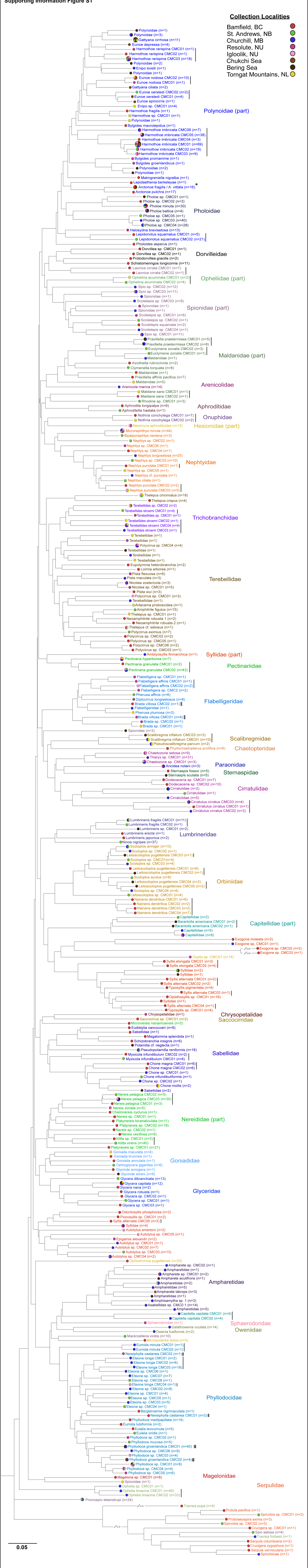

Figure S1. Neighbour-joining tree for 333 provisional polychaete species. One specimen per cluster is shown with abundances indicated in brackets. Collection locations are indicated by pie graphs. Vertical bars indicate identified species whose members fell into two or more MOTUs.
