# Supplementary material for: A Tri-Oceanic Perspective: DNA Barcoding Reveals Geographic Structure and Cryptic Diversity in Canadian Polychaetes
Source: PLoS One. 2011 Jul 14;6(7):e22232. doi: 10.1371/journal.pone.0022232 (PMC3136506; doi:10.1371/journal.pone.0022232)
Supplement: Table S1 — List of primers used in this study. (PDF) [file pone.0022232.s002.pdf]

Supporting Information Table S1. List of primers used in this study.

Primer name		Sequence (5' – 3')	Reference
polyLCO (F)		GAYTATWTTCAACAAATCATAAAGATATTGG	This study
polyHCO (R)		TAMACTTCWGGGTGACCAAARAATCA	This study
PolyshortCOIR (R)		CCNCCTCCNGCWGGRTCRAARAA	This study
C_VF1LFt1 (F)	Ratio		[42]
LepF1_t1	1	TGTAAAACGACGGCCAGTATTCAACCAATCATAAAGATATTGG	
VF1_t1	1	TGTAAAACGACGGCCAGTTCTCAACCAACCACAAAGACATTGG	
VF1d_t1	1	TGTAAAACGACGGCCAGTTCTCAACCAACCACAARGAYATYGG	
VF1i_t1	3	TGTAAAACGACGGCCAGTTCTCAACCAACCAIAAIGAIATIGG	
C_VR1LRt1 (R)			[42]
LepRI_t1	1	CAGGAAACAGCTATGACTAAACTTCTGGATGTCCAAAAAATCA	
VR1_t1	1	CAGGAAACAGCTATGACTAGACTTCTGGGTGGCCAAAGAATCA	
VR1d_t1	1	CAGGAAACAGCTATGACTAGACTTCTGGGTGGCCRAARAAYCA	
VR1i_t1	3	CAGGAAACAGCTATGACTAGACTTCTGGGTGICCIAAIAAICA	
