# Supplementary material for: A Tri-Oceanic Perspective: DNA Barcoding Reveals Geographic Structure and Cryptic Diversity in Canadian Polychaetes
Source: PLoS One. 2011 Jul 14;6(7):e22232. doi: 10.1371/journal.pone.0022232 (PMC3136506; doi:10.1371/journal.pone.0022232)
Supplement: Table S2 — Polychaete family richness and abundance by sampling site. (PDF) [file pone.0022232.s003.pdf]

Supporting Information Table S2. Polychaete family richness and abundance by sampling site.

Family	MOTUs	N	Number of Species								No. taxa with >1 MOTU
			ST	TOR	CHU	IGL	RES	CHK	BS	BAM	
Polynoidae	38	294	6	5	7	3	10	9	11	11	5
Phyllodocidae	30	164	5	1	13	3	5	1	2	5	4
Syllidae	28	86	2	-	6	2	3	-	2	15	2
Terebellidae	28	95	3	6	5	3	6	-	1	10	1
Spionidae	18	106	4	1	8	-	4	-	1	3	1
Orbiniidae	16	68	4	-	4	-	-	-	4	5	2
Sabellidae	15	60	3	1	3	-	1	-	2	7	2
Nereididae	14	150	2	-	1	2	1	-	1	10	2
Maldanidae	13	46	4	2	3	-	1	-	2	3	2
Nephtyidae	13	96	5	2	3	2	2	-	-	3	1
Flabelligeridae	12	30	1	2	1	1	6	-	-	1	2
Ampharetidae	11	38	1	-	8	-	1	-	2	1	
Cirratulidae	11	76	1	-	6	2	3	-	1	3	1
Pholoidae	8	111	3	-	4	-	2	-	3	2	
Capitellidae	7	24	1	-	4	-	1	-	2	-	2
Glyceridae	7	31	2	1	1	-	1	-	1	4	
Opheliidae	7	90	1	2	4	-	-	-	-	1	2
Goniadidae	6	19	1	1	-	-	-	-	1	3	
Lumbrineridae	6	54	2	1	1	-	-	-	1	2	1
Trichobranchidae	6	13	1	3	2	-	3	-	-	-	1
Serpulidae	9	15	2	-	1	-	-	-	-	6	
Dorvilleidae	4	15	-	-	-	-	-	-	-	4	
Hesionidae	4	54	-	-	-	1	2	-	-	2	
Pectinariidae	4	52	2	1	2	2	-	-	-	1	1
Scalibregmidae	3	15	-	2	2	-	-	-	1	-	1
Aphroditidae	2	7	1	-	-	-	-	-	-	1	
Onuphidae	2	9	-	1	-	-	-	-	-	1	1
Oweniidae	2	16	-	-	2	-	-	-	-	-	
Sternaspidae	2	7	-	-	-	-	-	-	1	1	
Arenicolidae	1	14	-	-	1	-	-	-	-	-	
Chaetopteridae	1	6	-	-	-	-	-	-	-	1	
Chrysopetalidae	1	1	-	-	-	-	1	-	-	-	
Magelonidae	1	8	-	-	-	-	-	-	-	1	
Paraonidae	1	3	-	-	1	-	1	-	-	-	
Saccocirridae	1	2	-	-	-	-	-	-	-	1	
Sphaerodoridae	1	1	-	-	-	-	1	-	-	-	
**Total**	**333**	**1876**	**57**	**32**	**93**	**21**	**55**	**10**	**39**	**108**	**34**

Families are listed in order of richness. Within each family, the abundance (N) and number of identified species containing multiple lineages are indicated. Regions (east to west) include: St. Andrews (ST); Torngat Mountains (TOR); Churchill (CHU); Igloolik (IGL); Resolute (RES); Chukchi Sea (CHK); Bering Sea (BS); and Bamfield (BAM).
